# The Brainstem in Emotion: A Review

**DOI:** 10.3389/fnana.2017.00015

**Published:** 2017-03-09

**Authors:** Anand Venkatraman, Brian L. Edlow, Mary Helen Immordino-Yang

**Affiliations:** ^1^Department of Neurology, University of Alabama at Birmingham, Birmingham, ALUSA; ^2^Department of Neurology, Massachusetts General Hospital and Harvard Medical School, Boston, MAUSA; ^3^Brain and Creativity Institute, University of Southern California, Los Angeles, CAUSA; ^4^Rossier School of Education, University of Southern California, Los Angeles, CAUSA; ^5^Neuroscience Graduate Program, University of Southern California, Los Angeles, CAUSA

**Keywords:** brainstem, emotion, networks, interoception, feeling, midbrain, pons, medulla

## Abstract

Emotions depend upon the integrated activity of neural networks that modulate arousal, autonomic function, motor control, and somatosensation. Brainstem nodes play critical roles in each of these networks, but prior studies of the neuroanatomic basis of emotion, particularly in the human neuropsychological literature, have mostly focused on the contributions of cortical rather than subcortical structures. Given the size and complexity of brainstem circuits, elucidating their structural and functional properties involves technical challenges. However, recent advances in neuroimaging have begun to accelerate research into the brainstem’s role in emotion. In this review, we provide a conceptual framework for neuroscience, psychology and behavioral science researchers to study brainstem involvement in human emotions. The “emotional brainstem” is comprised of three major networks – Ascending, Descending and Modulatory. The Ascending network is composed chiefly of the spinothalamic tracts and their projections to brainstem nuclei, which transmit sensory information from the body to rostral structures. The Descending motor network is subdivided into medial projections from the reticular formation that modulate the gain of inputs impacting emotional salience, and lateral projections from the periaqueductal gray, hypothalamus and amygdala that activate characteristic emotional behaviors. Finally, the brainstem is home to a group of modulatory neurotransmitter pathways, such as those arising from the raphe nuclei (serotonergic), ventral tegmental area (dopaminergic) and locus coeruleus (noradrenergic), which form a Modulatory network that coordinates interactions between the Ascending and Descending networks. Integration of signaling within these three networks occurs at all levels of the brainstem, with progressively more complex forms of integration occurring in the hypothalamus and thalamus. These intermediary structures, in turn, provide input for the most complex integrations, which occur in the frontal, insular, cingulate and other regions of the cerebral cortex. Phylogenetically older brainstem networks inform the functioning of evolutionarily newer rostral regions, which in turn regulate and modulate the older structures. Via these bidirectional interactions, the human brainstem contributes to the evaluation of sensory information and triggers fixed-action pattern responses that together constitute the finely differentiated spectrum of possible emotions.

## Introduction

Emotions are mental and bodily responses that are deployed automatically when an organism recognizes that a situation warrants such a reaction (Damasio, 1994). Due to humans’ intellectual capacities, human emotional reactions are not necessarily triggered by immediate (real) physical or social circumstances, but can also be precipitated by inferences, memories, beliefs or imaginings (Immordino-Yang, 2010). Although human emotions can involve complex cognitive deliberations (Immordino-Yang, 2010, 2015) their activating power fundamentally depends upon the modulation of arousal, motor control and somatosensation. Emotions are therefore regulated by a broad range of subcortical and cortical structures, with a critical role being played by subcortical nuclei in the pontine and midbrain tegmentum (Nauta, 1958; Parvizi and Damasio, 2001), as well as by autonomic and cardiorespiratory nuclei in the medulla (Edlow et al., 2016). Currently, most investigations of human emotion, especially in the neuropsychology literature, have focused on contribution of cortical rather than subcortical structures to human emotion, with a few notable exceptions (Buhle et al., 2013). Given that the brainstem plays a critical role in regulating and organizing emotion-related processing, the aim of this review is to provide a conceptual framework for affective researchers to study the brainstem’s role in human emotion.

## Organization of Brain Regions Involved in Emotion

**FIGURE 1:**
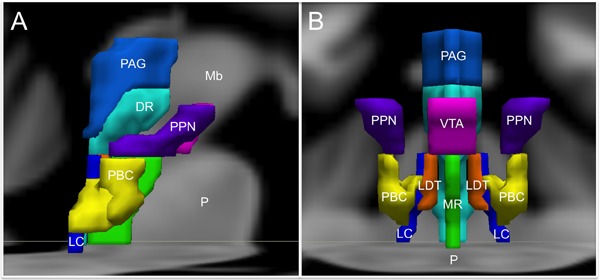
**Brainstem nuclei involved in human emotion. (A)** Sagittal view and **(B)** Coronal view. DR, Dorsal Raphe; LC, Locus coeruleus; LDT, Laterodorsal tegmental nucleus; Mb, Midbrain; MR, Median raphe; P, Pons; PAG, Periaqueductal gray; PBC, Parabrachial nuclear complex; PPN, Pedunculopontine nucleus; VTA, Ventral tegmental area. The substantia nigra and the nucleus of the tractus solitarius are not shown to optimize visibility of the other structures.

For the purpose of studying its role in emotion, the brainstem can be conceptualized as being composed of Ascending, Descending, and Modulatory networks. The gray matter nodes and white matter connections within each of these networks are summarized in **Table 1**, while **Figure 1** provides a schematic overview of the networks’ brainstem nodes. Our description of an Ascending sensory network in the brainstem that contributes to emotion is rooted in prior work by Parvizi and Damasio (2001) and Damasio and Carvalho (2013). The Descending network is based upon the “emotional motor system” initially proposed by Holstege (2009). The Modulatory network is based upon evidence showing that multiple brainstem-derived modulatory neurotransmitters contribute to emotion and emotional behavior (Alcaro et al., 2007; Berridge and Kringelbach, 2008; Dayan and Huys, 2009).

**Table 1:** The three networks of brainstem structures involved in emotion processing, and their components.

Network	Important structures
Ascending (sensory)	Spinothalamic tracts; Medial forebrain bundle; Nucleus of the tractus solitarius; Parabrachial nuclear complex; Thalamic nuclei
Descending (motor)	Lateral: periaqueductal gray and its projections Medial: Caudal raphe nuclei, locus coeruleus and their projections
Modulatory	Raphe nuclei (serotonergic) Locus coeruleus (noradrenergic) Ventral tegmental area (dopaminergic) Pedunculopontine and laterodorsal tegmental nuclei (cholinergic)

Integration of signaling within these three networks occurs at all levels of the brainstem, while progressively more complex levels of integration occur in the thalamus, hypothalamus and cerebral cortex. This encephalization and hierarchical organization allows phylogenetically older pathways in the brainstem, which evaluate sensory information and give rise to fixed-action pattern responses, to be regulated by evolutionarily newer rostral regions (Tucker et al., 2000). It is important to emphasize here that this conceptual model is based upon limited information about the functioning of the human brainstem, and will likely require revision and further differentiation as new evidence arises (Seeley et al., 2007; Coenen et al., 2011; Hermans et al., 2014).

## Ascending Network

Damasio’s (1996) Somatic Markers Hypothesis suggests that emotion processing incorporates somatosensory and visceral feedback from the periphery, either directly or through intervening sensory representations in caudal structures. Multiple representations of the body state in the brainstem and in the insular cortices are believed to enable simulation of future actions and sensations to guide decision making, as well as to contribute to empathy and theory of mind in humans. Self-awareness may arise from successive temporal representations of the body with increasing levels of detail (Craig, 2003a). Even the simple sensory representations of the body in the brainstem nuclei can alter affective experience, as demonstrated by studies showing that subtle modulation of a subject’s facial expressions can change self-reported affect (Harrison et al., 2010).

Interoception, which is the sense of the internal condition of the body, and emotional feeling, may share a common route through the brainstem to the anterior insular cortex (Craig, 2003a; Drake et al., 2010). The interoceptive system, represented in the cortex by the insula and adjacent regions of the frontal operculum, is particularly important for the internal simulation of observed emotion in humans (Preston et al., 2007; Pineda and Hecht, 2009) and for the experience of complex social emotions (Immordino-Yang et al., 2009, 2014, 2016). The other body map in the somatosensory cortex, which is built from dorsal column inputs and segments of the anterolateral pathway, contributes to affective understanding by simulation of facial expressions (Pineda and Hecht, 2009), analogous to the proposed function of primate mirror neurons in perception/action coupling (Rizzolatti and Craighero, 2004).

**FIGURE 2:**
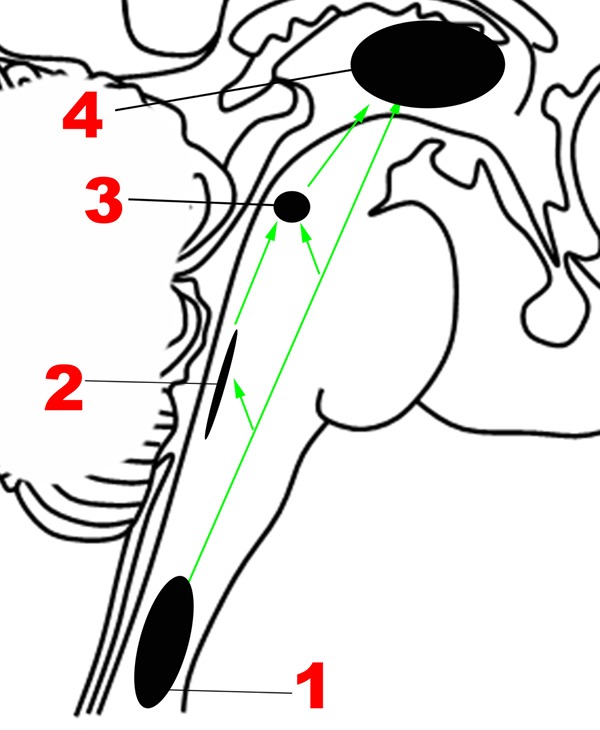
**Major structures involved in the Ascending network.** (1) Spinothalamic tracts. (2) Nucleus of the tractus solitarius. (3) Parabrachial nuclear complex. (4) Thalamus. Green arrows: Ascending projections.

The neuroanatomic basis for the Ascending sensory network and the mechanisms by which it modulates human emotion remain poorly understood. Although the structural and functional properties of these ascending pathways have been studied extensively in rodents and non-human primates using premortem tract-tracing and invasive electrophysiological studies, these techniques cannot be applied in humans. Recent studies using diffusion tractography and resting-state functional connectivity techniques in humans have found that forebrain regions involved in regulation of mood and affect are interconnected not only with mesencephalic and pontine arousal nuclei, but also with medullary cardiorespiratory and autonomic nuclei through the medial and lateral forebrain bundles (Vertes, 2004; Edlow et al., 2016). **Figure 2** provides an overview of the main structures in the Ascending network.

It is well established that sensations from the human body are carried in two major ascending pathways in the brainstem – the dorsal columns of the spinal cord, which continue as the medial lemnisci, carry discriminatory sensation, deep touch and proprioception; the anterolateral pathway, composed of the spinothalamic tracts, carries nociceptive and temperature-related signals (Nogradi et al., 2000-2013).

### The Anterolateral Pathway

The nociceptive fibers in the anterolateral pathway give off collaterals at every level that converge with projections from visceral sensory neurons in the brainstem, thereby ensuring close coordination of pain and autonomic processing (Craig, 2003b). The pathway begins with small-diameter fibers that transmit signals of fast and slow pain, chemical changes, temperature, metabolic state of muscles, itch, and sensual or light touch to lamina I of the spinal cord, from where ascending projections arise. In the caudal brainstem, these projections target the nucleus of the tractus solitarius in the medulla (**Figure 2**), which is also innervated by visceral and taste sensations through the vagus, glossopharyngeal and facial nerves.

### The Parabrachial Complex

Tract-tracing studies in rodent models have revealed that ascending projections from the nucleus of the tractus solitarius travel to the parabrachial complex (**Figures 1**, **2**) in the upper pons (Herbert et al., 1990), which also receives direct projections from lamina I neurons (Craig, 2003b), in addition to other inputs such as balance (Balaban, 2002). Rat studies suggest that the parabrachial complex integrates multiple types of converging sensory inputs and in turn projects to rostral regions such as the thalamus, hypothalamus, basal forebrain and amygdala, and may play an important role in arousal (Fuller et al., 2011; Edlow et al., 2012). The upper brainstem, where the parabrachial complex lies, is therefore the most caudal structure where a topographically complete map of the body can be assembled that includes all manner of interoceptive information (Damasio and Carvalho, 2013). There is also ongoing investigation of the role played by the superior colliculus, a structure in the dorsal aspect of the upper brainstem, in sensory and emotional processing in humans, but the available evidence is sparse (Celeghin et al., 2015).

### The Thalamus

Immediately rostral to the upper brainstem is the thalamus, and the spinothalamic tracts, as their name indicates, end in the thalamus. A subset of thalamic nuclei function as relay structures between the emotional brainstem and rostral brain structures. The ventral posteromedial nuclei of the thalamus, which receive projections from the parabrachial complex and other parts of the anterolateral pathway, project to the insular cortex, particularly the mid/posterior dorsal part. Craig and colleagues suggested that the posterior part of the ventral medial nucleus of the thalamus, or VMPo, was uniquely involved in pain processing, particularly in primates (Craig, 2003a), but other authors had questioned the separate existence of this nucleus (Willis et al., 2002).

The intralaminar nuclei of the thalamus receive non-topographical sensory input from the spinal cord, which are in turn projected to the orbitofrontal and anterior cingulate cortices. The intralaminar nuclei are involved in orienting and attention, while arousal and visceral sensation are subserved by the midline nuclei (Morgane et al., 2005). In primates a direct pathway from lamina I to the anterior cingulate through the medial dorsal nucleus is also present (Craig, 2003a), and it has been suggested that these pathways may mediate the affective aspect of pain (Tucker et al., 2005). Indeed, the mediodorsal nucleus progressively increases in cytoarchitectonic complexity in higher animals, and is also known to project to the frontal and prefrontal cortices (Morgane et al., 2005). Thus, the thalamus contains multiple structures that appear to play a role in transmitting the signals essential for emotion processing from the brainstem to the forebrain.

Summary statement: Representations of the body of varying degrees of complexity that exist at multiple levels in the Ascending network, including the nucleus of the tractus solitarius and the parabrachial nucleus, are believed to be give rise to the “feeling” of an emotion.

## Descending Network

**FIGURE 3:**
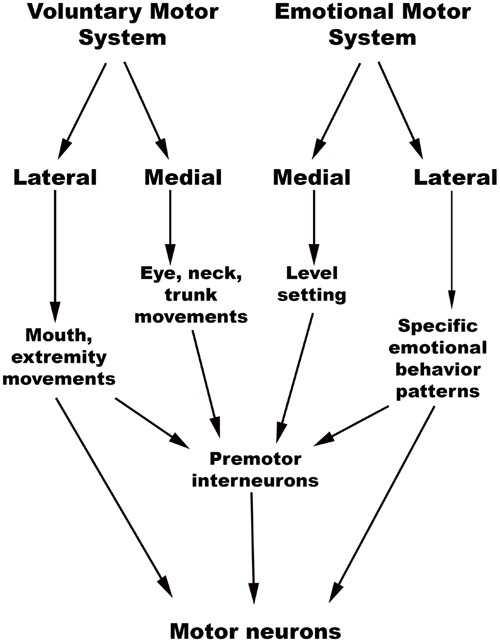
**Holstege’s conception of the Emotional and Somatic motor systems. (Adapted from Holstege, 2016)**.

The chief descending pathway in the human brainstem is composed of large, myelinated axons of the corticospinal tracts, transmitting motor impulses to the anterior horn cells of the spinal cord and thereafter to skeletal musculature (Nogradi and Gerta, 2000–2013). In addition, the midbrain and pontine tegmentum, as well as the medulla, contain several structures that serve as the output centers for motor and autonomic regulatory systems, which in turn regulate the bodily manifestations of the “emotion proper” (Damasio, 1994). Holstege (2009) considered the interconnected network of descending fibers and effector regions in the brainstem an “emotional motor system,” distinct from the corticospinal somatic motor pathway, each of which they divided into lateral and medial parts [**Figure 3**, adapted from (Holstege, 2016)].

The brainstem, as noted previously, contains a hierarchy of circuits linking ascending sensory neurons and descending effector neurons. Evidence from rat and cat studies indicates that the lower-level circuits enable quick stereotypical responses to stimuli, while the higher-level involvement of rostral centers allows for complex motor and autonomic activity and action specificity (Bandler et al., 2000; Gauriau and Bernard, 2002). This close relationship between sensory and effector networks in emotion processing is best illustrated by the close overlap seen between sites involved in emotional vocalization and pain processing in animals. Both physical and psychological pain (caused by separation from caregivers, for example) can produce distress vocalizations in animals, with the caudal brainstem containing multiple regions that control the respiratory and phonetic changes of vocalization (Tucker et al., 2005) and cardiorespiratory function during emotion (Lovick, 1993; Rainville et al., 2006; Edlow et al., 2016). The rostral nuclei are able to modulate the activity of caudal nuclei that control cardiorespiratory control and vocalization in a coordinated manner that makes the resultant action more complex and nuanced.

### Lateral Part of the Emotional Motor System

**FIGURE 4:**
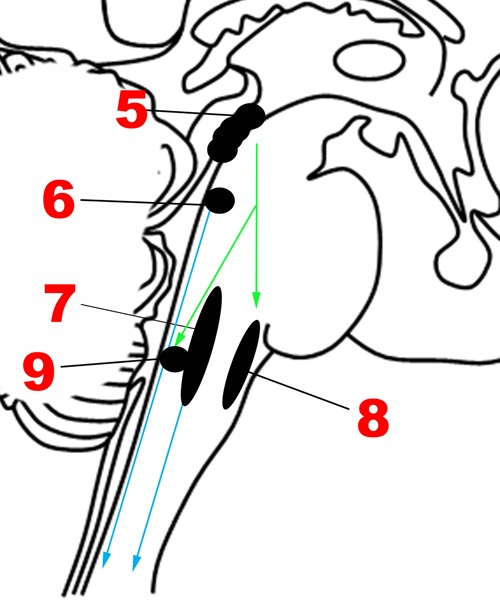
**Major structures involved in the Descending network**. (5) Periaqueductal gray. (6) Locus coeruleus. (7) Caudal raphe nuclei. (8) Rostral ventrolateral medullary nuclei. (9) Dorsal motor nucleus of the vagus nerve. Green arrows: Descending projections from periaqueductal gray. Blue arrows: Descending projections from the caudal raphe and locus coeruleus.

The emotional motor system’s lateral part consists of projections primarily from the periaqueductal gray, as well as more rostral structures such as the amygdala and hypothalamus, to the lateral tegmentum in the caudal pons and medulla (**Figures 3**, **4**). This lateral part of the emotional motor system is involved in specific motor actions invoked in emotions, as well as in the control of heart rate, respiration, vocalization, and mating behavior (Holstege, 2009). Studies in multiple animal models as well as in humans have revealed that the periaqueductal gray (**Figures 1**, **4**) is a major site of integration of affective behavior and autonomic output, with strong connections to other brainstem structures (Behbehani, 1995).

Several fixed patterns of behavior, particularly those related to responding to external threats, with accompanying autonomic changes, are organized in the different columns of the periaqueductal gray in rats (Brandao et al., 2008). The lateral/dorsolateral column receives well-localized nociceptive input (superficial ‘fast’ pain, as might be expected from bites or scratches) and is believed to organize fight-or-flight reactions. When stimulated this column produces emotional vocalization, confrontation, aggression and sympathetic activation, shown by increased blood pressure, heart rate, and respiration. Many of these responses are mediated by descending projections to the paragigantocellularis lateralis nucleus in the rostral ventrolateral medulla (respiratory rhythm), the dorsal motor nucleus of the vagus (heart rate and rhythm), and caudal raphe (cardiorespiratory integration; Lovick, 1993; Edlow et al., 2016). Within this dorsolateral/lateral column itself, there are two parts. The rostral part is responsible for power/dominance (producing a “fight” response), while the caudal part invokes fear (producing a “flight” response) with blood flow to the limbs (Sewards and Sewards, 2002).

The ventrolateral column of the periaqueductal gray receives poorly localized “slow, burning” somatic and visceral pain signals, and on stimulation produces passive coping, long-term sick behavior, freezing with hyporeactivity and an inhibition of sympathetic outflow (Parvizi and Damasio, 2001; Craig, 2003b; Brandao et al., 2005; Benarroch, 2006). In this way, it is likely involved in background emotions such as those that contribute to mood. Rat studies have further revealed that lesions of the dorsolateral periaqueductal gray reduce innate defensive behaviors, while lesions of the caudal ventrolateral part reduce conditioned freezing and increase locomotor activity (Brandao et al., 2005). When the predator is far away, the ventromedial prefrontal cortex and the hippocampus, through the amygdala, activate midbrain structures centered around the ventrolateral periaqueductal gray, which results in freezing (Tucker et al., 2000). In the “circa-strike” stage when the predator is imminent, forebrain pathways are silenced, and the dorsolateral periaqueductal gray is activated, resulting in fight-or-flight reactions.

### The Periaqueductal Gray in Human Emotion

Though the reactions detailed above are almost certainly incorporated into human emotion, the precise mechanisms have not been elucidated. One study involving high-resolution MRI of the human periaqueductal gray indicated that this structure has discrete functional subregions that parallel the divisions seen in animals – aversive stimuli caused activation in the ventrolateral regions of the caudal periaqueductal gray and in the lateral/dorsomedial regions of the rostral periaqueductal gray (Satpute et al., 2013). The periaqueductal gray threat response system is likely co-opted in the pathophysiology of conditions such as panic disorder and generalized anxiety disorder. Blood flow analysis suggests that the inhibitory influence of the cortex over the fight-or-flight mechanisms in the periaqueductal gray is reduced in panic disorder (Del-Ben and Graeff, 2009). Functional MRI has also revealed activation of the human periaqueductal gray in complex emotions such as frustration (Yu et al., 2014), admiration and compassion (Immordino-Yang et al., 2009), in addition to more immediate threat responses (Lindner et al., 2015).

### Medial Part of the Emotional Motor System

The medial part of the emotional motor system (**Figures 3**, **4**) consists of descending projections from the reticular formation that are involved in level-setting and modulatory functions (Holstege, 2009). Once again, the vast majority of the research on this subject has been in animals. The caudal third of the locus coeruleus (Sasaki et al., 2008) and the caudal raphe nuclei both send projections downward to the spinal cord, as depicted in **Figure 4**, and are responsible for descending pain modulation (Renn and Dorsey, 2005). The effect of norepinephrine from the locus coeruleus is mostly antinociceptive, while serotonin from the raphe nuclei can have varying effects depending upon the type of receptor activated (Benarroch, 2008). In rats, it has been shown that the midbrain tectum and the dorsal/lateral periaqueductal gray indirectly produce the analgesia that occurs in fear (Coimbra et al., 2006), through a primarily non-opioid mechanism involving GABAergic and serotonergic neurons (as opposed to the ventrolateral periaqueductal gray that produces a long-lasting opioid mediated analgesia; Gauriau and Bernard, 2002). It is likely that this system of fear suppressing the pain system is still present in humans, allowing us to act and move rapidly in situations of threat (Mobbs et al., 2007).

In addition to nociceptive modifications, the medial part of the emotional motor system is also involved in level-setting for arousal levels and muscle function – studies on rodents and monkeys indicate that this is accomplished through norepinephrine secretion from the locus coeruleus (Aston-Jones and Cohen, 2005; Lang and Davis, 2006) and cholinergic projections from the pedunculopontine tegmental nucleus in the upper pons (Bechara and van der Kooy, 1989; Homs-Ormo et al., 2003). Further detail regarding these important structures is provided in the section below on the Modulatory network.

Summary statement: The Descending network, otherwise referred to here as the emotional motor system, has a lateral part that triggers patterned emotional behaviors, while the medial part is responsible for level-setting in sensory and arousal systems that might be important in emotionally charged situations.

## Modulatory Neurotransmitter Network – Valence, Arousal, and Reward

**FIGURE 5:**
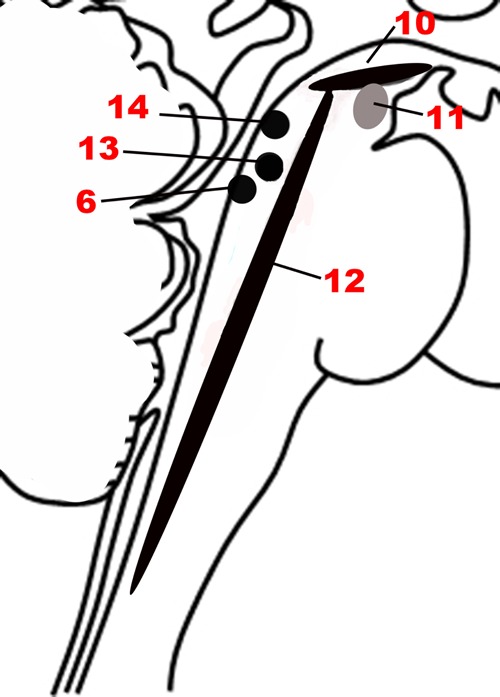
**The nuclei of the Modulatory network.** (10) Substantia nigra. (11) Ventral tegmental area. (12) Raphe nuclei. (6) Locus coeruleus. (13) Pedunculopontine nucleus. (14) Laterodorsal tegmental nucleus.

Since a major characteristic of an adaptive emotional behavioral response is flexibility, a network that modulates the autonomic, motor, affective and memory changes brought about by different stimuli is needed. The chief upper brainstem structures involved in this modulation are the neurotransmitter pathways arising from the upper raphe nuclei (serotonergic), the ventral tegmental area-substantia nigra pars compacta complex (dopaminergic), and the upper locus coeruleus (noradrenergic), which project widely throughout the hypothalamus, cortex and other parts of the forebrain. In addition, the laterodorsal and the pedunculopontine tegmental nuclei are sources of cholinergic fibers, which stimulate cortical activation through the thalamus. These structures are depicted in **Figures 1**, **5**. Ascending projections from the brainstem to subcortical and cortical structures communicate the states of brainstem structures to more rostral regions of the nervous system, where these states contribute to affective experience. Since these pathways are involved in arousal and in the maintenance of consciousness (Jones, 2003), they are sometimes called the Ascending Reticular Activating System or Ascending Arousal Network (Moruzzi and Magoun, 1949; Edlow et al., 2012). The following sections on the various pathways that comprise the Modulatory network are in large part descriptions of the Ascending Reticular Activating System, albeit with a focus on how these relate to emotion.

### The Valence-Arousal Model of Emotion and Its Critiques

The modulation of affective states by these upper brainstem-based pathways has been expressed through the two domains of valence and arousal. According to the circumplex model of emotions, each basic emotion is postulated to be a combination of these two domains, in differing degrees (Russell, 1980; Zald, 2003; Posner et al., 2009). In humans, valence correlates with pleasantness ratings, heart rate, and facial muscle activity, while arousal correlates with skin conductance, interest ratings and viewing time for stimuli (Lang and Davis, 2006). Both valence and arousal have significant impact on an organism’s relationship with the environment, influencing, for example, the allocation of attention and long term memory formation (Arbib and Fellous, 2004).

Recent work, especially in the neuroimaging literature, has raised questions about whether complex neurological processes like emotions can actually be represented by reducing to dimensions of valence and arousal. Kragel and LaBar (2016), in an interesting review of the nature of brain networks that subserve human emotion, argue that each emotion uniquely correlates with activation of a constellation of cortical and subcortical structures (Kragel and LaBar, 2016), and that the current neuroimaging data do not support the valence-arousal model of emotions. They focused on fMRI studies which have applied novel statistical methods collectively known as multivoxel pattern analysis to identify mappings between mental states and multiple measures of neural activity. The mainstay of earlier neuroimaging research on emotion was univariate pattern analysis, but multivariate analyses have the advantages of higher sensitivity, and the ability to detect counterintuitive relationships because of the lack of reliance on *a priori* hypotheses. These approaches also have the advantage of overcoming the assumption that dedicated modules or homogeneous neural units subserve each emotion, because they can investigate various neuronal populations at much larger spatial scales.

Kragel and LaBar (2016) suggest that while the use of machine learning approaches to large neuroimaging datasets is likely to expand in the near future, it might be premature to draw conclusions about neural substrates underlying each emotion, because the current studies using multivariate analyses have not all been consistent with one another. These differences may be coming from technical variations in the methods used to induce and assess the emotion and associated neural activations, but might also represent fundamental variations in the circuitry employed in different individuals, or even a lack of emotional “essences” that can be studied in a standardized manner across people and cultures. While this is a valid critique, we believe that the older valence-arousal classification still holds value in furthering our understanding of brainstem contributions to emotions and especially to basic emotions shared with intelligent animals. This debate may eventually be resolved with technical advances in functional neuroimaging and multidisciplinary approaches to studying emotional experiences (Immordino-Yang and Yang, 2017, in press).

## Dopamine and Reward Pathways

Emotional valence is closely tied to the concept of reward and punishment (Dayan and Huys, 2009). Rewards, both natural (such as that induced by social play in animals) and drug-induced, include both hedonic and motivational aspects (Trezza et al., 2010). While the core hedonic status, as demonstrated by consummatory pleasure and facial expressions, is believed to be primarily based on opioid transmission, motivation is more dependent on dopamine transmission (Alcaro et al., 2007). The separation between the motivation and liking systems may have allowed the same motivational circuitry to be used in positive and negative events (Berridge and Robinson, 2003).

### Anatomy

Brainstem dopaminergic neurons in mammals are located in the midbrain, and are typically divided into three contiguous groups: the retrorubral field, the substantia nigra pars compacta, and the ventral tegmental area (**Figures 1**, **5**). There are two major ascending dopaminergic pathways arising from these clusters: the nigrostriatal pathway from the substantia nigra pars compacta, and the mesocorticolimbic pathway from the ventral tegmental area (Arias-Carrion and Poppel, 2007). The medially situated mesocorticolimbic pathway is evolutionarily older than the laterally situated nigrostriatal pathway (Alcaro et al., 2007). The dopaminergic neurons of the ventral tegmental area are subject to feedback inhibition from the cortex and the striatum. The pedunculopontine nucleus also sends ascending projections that have been shown to affect tonic dopamine release and arousal in studies on rats and monkeys (Sesack et al., 2003; Xiao et al., 2016).

### Function

The mesocorticolimbic pathway is thought to be part of a larger, general-purpose appetitive foraging system in animals that enables establishment of adaptive expectations about the configurations and reward-availability in the environment, with dopamine inducing a “seeking” disposition toward the environment (Alcaro et al., 2007). This seeking disposition itself may have hedonic properties independent of reward attainment. Studies on rats and other models indicate that dopaminergic neurons in the ventral tegmental area show both tonic activity, maintaining a baseline level of dopamine in the brain, and phasic burst firing in response to certain cues (Grace, 1991). The two are believed to antagonize each other (Ikemoto, 2007). Unpredicted rewards, prediction errors (Song and Fellous, 2014), novel stimuli (Bunzeck and Duzel, 2006), physically salient stimuli, motivational/affective salience, and attention shifts related to approach behavior are all potential causes of altered dopaminergic firing based on studies in rats, monkeys and humans, although only a subpopulation of the neurons in the ventral tegmental area may be activated in each case (Schultz, 2010). Tonic dopamine release, on the other hand, promotes arousal in almost all mammals, and this is likely achieved by D2-receptor-mediated inhibition of cortical and limbic top-down control over subcortical structures (Alcaro et al., 2007; Song and Fellous, 2014).

An appetitive/aversive opponency is thought to exist between the serotonin and dopamine pathways, with serotonin antagonizing several energizing and appetitive effects of dopamine (Dayan and Huys, 2009). One series of experiments on rats showed that single bursts of norepinephrine release from the locus coeruleus activated dopaminergic firing (Grenhoff et al., 1993), but in depressive mood states, sustained burst firing of locus coeruleus neurons was seen, which caused suppression of dopamine release (Grenhoff et al., 1993; Weiss et al., 2005). Significant attention has been focused on the role of dopamine in the motivational deficits seen in depression, schizophrenia, Parkinson’s disease and other disorders (Salamone et al., 2016), and the antidepressant bupropion thought to exert its effects through inhibition of the reuptake of both norepinephrine and dopamine (Patel et al., 2016).

Summary statement: Dopaminergic neurons from the ventral tegmental area show both tonic and phasic firing patterns, are involved in reward, motivation, and arousal, and malfunction of these pathways likely contributes to motivational deficits in depression.

## Serotonergic Pathways and the Raphe Nuclei

### Anatomy

The cell bodies of all the serotonergic neurons in the human brain lie in the raphe nuclei (**Figures 1**, **5**). They are clustered along the midline throughout the brainstem. The rostral group lies in the midbrain and upper pons (caudal linear, dorsal raphe, and median raphe nuclei), while the caudal group lies in the lower pons and medulla (raphe magnus, raphe obscurus, and raphe pallidus nuclei). The rostral raphe nuclei mainly send ascending projections, while the caudal raphe send descending projections as discussed above (Hornung, 2003).

### Function

Serotonin modulates the sensitivity of the fear/defense circuitry and the magnitude of these responses in response to various stimuli. Inescapable shock, for instance, produces inhibition of the fight-flight defensive response and activation of the fear-anxiety response in rats (Maier and Watkins, 2005). This might be through its suppression of panic and escape reactions encoded in the dorsal periaqueductal gray (Zangrossi et al., 2001). Serotonin is also involved in regulation of social behaviors such as aggression, status-seeking and affiliation (Arbib and Fellous, 2004; Gobrogge et al., 2016). It is believed to enable prosocial and agreeable behavior in humans as well as other animals (Moskowitz et al., 2003). A correlation between anxiety, depression and serotonin is suggested by the effectiveness of Selective Serotonin-Reuptake Inhibitor (SSRI) drugs in mood disorders (Adell, 2015). The dorsomedial part of the dorsal raphe is particularly important for anxiety-related processing in humans, receiving innervations from several forebrain structures implicated in anxiety, including the bed nucleus of the stria terminalis (Lowry et al., 2008).

It must be noted that studies on the role of serotonin in affective control have yielded contradictory results (Dayan and Huys, 2009). Though serotonin projections to the amygdala enhance anxiety, those to the hippocampus are associated with depression and the retrieval of fear memories, and are known to contribute to hyperalgesic effects in times of stress (Dayan and Huys, 2009; Ohmura et al., 2010). One possibility, as noted by Gold (2015), is that the level of arousal may be an important factor in determining how abnormalities in serotonergic signaling manifest themselves. The chief distinction between melancholic or typical depression and atypical depression is that the former is worst in the morning, when arousal systems are at their maxima, while the latter is the worst in the evenings, when arousal systems are winding down (Gold, 2015). Another explanation is that the tremendous diversity in the types of serotonin receptors allows it to exert varying effects in relation to emotion and mood (Meneses and Liy-Salmeron, 2012).

Summary statement: Serotonin from the raphe nuclei appears to perform different functions in anxiety, stress, depression, and social behavior, likely because it acts through a diverse set of receptors, and its role may vary with the level of arousal.

## Norepinephrine and the Locus Coeruleus

### Anatomy

Studies in the monkey have revealed that the locus coeruleus (**Figures 1**, **4**, **5**) is innervated by the amygdala, anterior cingulate and orbitofrontal cortices, which are rostral centers involved in evaluating the motivational significance of a stimulus, as well as the raphe, which transmit viscerosensory stimuli from the nucleus of the tractus solitarius (Aston-Jones and Waterhouse, 2016). Thus the locus coeruleus, which is activated by stress, can integrate both external sensory and visceral signals and influence several effector targets, including the arousal pathways and the adrenal medulla (Sara, 2009).

### Function

The role of norepinephrine is understood to be twofold. It maintains a basal level of neuronal activity in the forebrain for the acquisition of sensory input (alertness), and also contributes to the level-setting in circuits involved in gathering and processing of salient, emotionally relevant information in both humans and animals (van Stegeren, 2008; Espana et al., 2016). Additionally, monkey experiments have indicated that norepinephrine and dopaminergic pathways may play a synergistic role in learning (Aston-Jones and Cohen, 2005). Studies on monkeys and fMRI investigations on humans suggest that arousal levels, primarily as determined by norepinephrine signaling, may gate learning, determining which events are prioritized for encoding in memory, and which are allowed to be forgotten (Mather and Sutherland, 2011). Beta-adrenoceptors appear particularly important in suppressing memory for less emotionally salient stimuli, and there may be an interaction of the norepinephrine signaling with sex hormones, especially in women (Clewett, 2016).

Summary statement: Norepinephrine pathways from the locus coeruleus are important in maintaining arousal, and also in level-setting for gathering sensory information and storing emotional memories.

## Cholinergic Pathways in the Brainstem

### Anatomy

The pedunculopontine nucleus and the laterodorsal tegmental nucleus (**Figures 1**, **5**) are the main cholinergic cell groups in the human brainstem. They provide the major cholinergic innervations of the thalamic relay nuclei and the reticular nucleus of the thalamus (Mesulam, 1995; Saper et al., 2005). Cortical structures, on the other hand, receive cholinergic innervations from cell groups outside the brainstem, in the basal forebrain (Mesulam, 2004).

### Function

Hyperpolarization of the GABAergic neurons in the reticular nucleus of the thalamus by cholinergic projections from the brainstem ultimately results in disinhibition of thalamic nuclei, and thereby influence level of arousal by gating of connections between the thalamic relay nuclei and cortical regions (Mesulam, 1995; Saper et al., 2005). Brudzynski (2014) suggests, based on anatomical studies in cats and rats that the ascending cholinergic projections from the laterodorsal tegmental nucleus to the forebrain and diencephalon form a mesolimbic pathway related to aversive emotional states, parallel to the mesocorticolimbic dopamine pathway that plays a role in motivation and positively valenced states. Stimulation of this pathway has been shown to produce an aversive response with distress vocalizations in these animal models, suggesting that this pathway serves as a “physiological, psychological, and social arousing and alarming system.” The pedunculopontine and laterodorsal nuclei have also been found to project extensively to the ventral tegmental area and substantia nigra pars compacta in a rat model, and are involved in reward processing through their effects on the dopaminergic pathways (Xiao et al., 2016).

Summary statement: The poorly studied cholinergic pathways in the brainstem are part of the ascending reticular activating system, and are thought to influence emotion primarily through their modulation of dopaminergic signaling.

## Other Transmitters in Emotion

GABAergic mechanisms serve to limit the arousal caused by the neurons of the Ascending Reticular Activating System (Lu and Greco, 2006). Histamine from the tuberomamillary nucleus in the hypothalamus is believed to increase neocortical arousal, and is involved in the modulation of other neurotransmitter pathways (Brudzynski, 2014). Hypocretin/orexin neurons arising from the hypothalamus project widely to various targets, including the limbic areas, and apart from modulating arousal, animal studies have also implicated them in fear and anxiety, reward processing (through projections to the Ventral Tegmental Area) and stress (Flores et al., 2015). Endorphins, endocannabinoids, and oxytocin are some other transmitter pathways known to play a role in emotion and valence. These pathways are not considered in greater detail here since their major source structures do not localize primarily to the brainstem tegmentum.

## Conclusion and Future Directions

The brainstem contains several structures that are likely of critical importance in the generation and experience of emotion. Most prior research on human emotion has focused on cortical mechanisms, largely because of the complexity of the brainstem coupled with the difficulty of analyzing brainstem functioning using current technologies. We have provided a conceptual overview of how tegmental structures of the brainstem are involved in emotion-related processes. Future research on the structural and functional connectivity of the human brainstem is needed to further understand its role in emotion. Such work will undoubtedly contribute to a more enriched and nuanced understanding of the neurobiology of human emotion in psychology and in affective neuroscience.

## Author Contributions

Conceptualization was by AV, BE, and MHI-Y. AV contributed to writing and framing the manuscript. Critical revision and guidance was provided by BE and MHI-Y. Figures were made by AV and BE.

## Conflict of Interest Statement

The authors declare that the research was conducted in the absence of any commercial or financial relationships that could be construed as a potential conflict of interest.
